# Quantifying Nurse and Physician Clinical Performance for Mechanically Ventilated Patients

**DOI:** 10.1111/1475-6773.70153

**Published:** 2026-07-29

**Authors:** Christopher F. Chesley, Yingying Lu, Rachel Kohn, Hayley B. Gershengorn, Kelly C. Vranas, Catherine L. Hough, Olga Yakusheva, Deena Kelly Costa, Deanna J. Marriott, David A. Asch, Aerielle Belk, Stefania Scott, Wei Wang, Meeta Kerlin

**Affiliations:** ^1^ Division of Pulmonary, Allergy, and Critical Care, Palliative and Advanced Illness Research (PAIR) Center, Department of Medicine, Leonard Davis Institute of Health Economics University of Pennsylvania Philadelphia Pennsylvania USA; ^2^ Palliative and Advanced Illness Research (PAIR) Center University of Pennsylvania Perelman School of Medicine Philadelphia Pennsylvania USA; ^3^ Division of Pulmonary, Critical Care, and Sleep Medicine University of Miami Miller School of Medicine Miami Florida USA; ^4^ Division of Critical Care Medicine Albert Einstein College of Medicine Bronx New York USA; ^5^ Division of Pulmonary, Allergy, & Critical Care Medicine Oregon Health & Science University Portland Oregon USA; ^6^ Center to Improve Veteran Involvement in Care and Section of Pulmonary & Critical Care Medicine VA Portland Health Care System Portland Oregon USA; ^7^ Department of Medicine Oregon Health & Science University Portland Oregon USA; ^8^ School of Nursing Johns Hopkins University Baltimore Maryland USA; ^9^ Yale School of Nursing Yale University New Haven Connecticut USA; ^10^ Section of Pulmonary, Critical Care and Sleep Medicine Yale School of Medicine New Haven Connecticut USA; ^11^ School of Nursing University of Michigan Ann Arbor Michigan USA; ^12^ Leonard Davis Institute of Health Economics, The Wharton School, Division of General Internal Medicine University of Pennsylvania Perelman School of Medicine Philadelphia Pennsylvania USA; ^13^ The Parity Center University of Pennsylvania Perelman School of Medicine Philadelphia Pennsylvania USA

**Keywords:** clinician performance, disease severity scores, mechanical ventilation, value‐added modeling

## Abstract

**Objective:**

Expanding on previous research applying value‐added modeling to clinician performance measurement, we quantified simultaneous nurse and physician performance among mechanically ventilated patients.

**Study Setting and Design:**

In a retrospective cohort spanning five academic hospitals, we used multiple linear regression to determine relationships between nurse and physician assignments and changes in patient illness severity (Laboratory‐based Acute Physiology Score) during intensive care unit (ICU) admission. Individual clinician performance was derived from regression coefficients. Collective clinician contribution to disease severity variance was assessed by comparing the coefficient of determination across clinician‐only and joint‐clinician models. Consistency of clinician performance estimates was cross‐validated using random 1:1 partitions of the patient population using Pearson's correlation.

**Data Sources and Analytic Sample:**

Electronic health record data were extracted from mechanically ventilated patients at study ICUs between 2018 and 2022.

**Principal Findings:**

Among 17,082 clinical encounters, 215 physicians, and 1719 nurses, nurse and physician assignments improved model fit. Together, they attributed 7% of variability in disease severity trajectory; separately, nurse assignments accounted for greater variance than physician assignments (6% vs. 4%, respectively). Cross‐partition clinician performance was moderately correlated (*r* = 0.31 [nurses]*, r* = 0.4 [physicians], *p* < 0.001 for both comparisons).

**Conclusions:**

Individual nurse and physician care independently contribute to disease severity trajectory among mechanically ventilated patients.

## Call Out Box

- What is known on this topic:
  ○ Interprofessional team‐based care is a guideline‐recommended delivery model for patients receiving mechanical ventilation; however, the attributable contributions of specific roles among interdisciplinary teams have yet to be characterized.
  ○ Value‐added modeling is a validated econometric technique that can quantify the performance of workers involved with a process and has previously been used to determine relationships between nurse performance and illness trajectory in hospitalized patients.
- What this study found:
  ○ Clinician‐specific value‐add based on assigned patients' illness trajectories can be reliably and independently quantified for nurse and physician roles in the context of acute hospital care for mechanically ventilated patients.
  ○ Illness trajectory may be more strongly associated with variability associated with nurse as opposed to physician care.

## 1 Introduction

Interprofessional team‐based care is a widely adopted [1, 2] and guideline‐recommended [3] critical care delivery model. Interprofessional teams leverage diversity of perspectives, knowledge, and skillsets [4]. While adoption [5, 6] of interprofessional teams and familiarity [7, 8] among team members are associated with reduced mortality, additional elements of interprofessional teams that drive clinical impacts are unknown.

Individual physicians are independently associated with mortality risk for mechanically ventilated patients [9] Within interprofessional teams, coordination of and collaboration between physician‐nurse dyads are also associated with clinical outcomes [10, 11]. However, it is unclear how and whether the contributions of nurses and physicians operate independently after adjusting for each role's' contributions. Clarifying these relationships may inform interprofessional team structure and dynamics to improve patient outcomes.

Value‐added modeling (VAM) is an econometric method that quantifies the independent performance of individual workers (or groups of workers) who contribute to the trajectory of a measurable outcome. The size and direction (positive or negative) of these independent associations determine the worker's value‐add relative to other workers. Worker performance can then be inferred based on value‐add relationships to the accomplishment of tasks. VAM has been most commonly deployed to measure individual teachers' relative contributions to educational test scores [12], but has also been applied to measure nurse contributions to the trajectory of illness severity among general medical‐surgical patients [13, 14, 15]. However, no prior VAM study has considered the simultaneous roles of both nurses and physicians, nor the context of critical illness. In this study, we use VAM to quantify the performance of nurses and physicians individually and the collective contribution of clinician groups using value‐add estimates derived from the change in patient disease severity throughout intensive care unit (ICU) admission.

## 2 Methods

The study was approved with a waiver of informed consent by the institutional review board at the University of Pennsylvania. We follow the Strengthening the Reporting of Observational Studies in Epidemiology (STROBE) reporting guidelines. Analyzes were performed between fall 2024 and fall 2025.

### 2.1 Study Sites and Population

The study included adults ≥ 18 years who were admitted to 14 ICUs at five hospitals between April 1, 2018, and October 31, 2021, and who received ≥ 12 h of mechanical ventilation. For patients with multiple episodes of mechanical ventilation during the same hospital encounter, only the first eligible episode was included. Patients were excluded if they had orders for comfort care at the time of ICU admission and if admission age was recorded as greater than 110 years, as this likely reflected indeterminable age for patients presenting without identifying information. ICUs included medical ICUs, surgical ICUs, mixed general medical and surgical units, and specialty ICUs such as neurological and cardiovascular ICUs. One hospital was a quaternary care urban academic medical center, two were urban referral hospitals, and two were community‐based suburban hospitals.

### 2.2 Data Sources and Study Variables

All study data were extracted from the integrated electronic health record (EHR) of the health system and included structured patient‐level data recorded during routine clinical operations. Enrollment for each patient began at the time of mechanical ventilation in the ICU and ended at the time of discharge from the ICU (including, if applicable, following the end of mechanical ventilation), either by transfer to another unit, discharge from the hospital, or death. We defined an ICU day as starting at 7:00 am and ending at 6:59 am the following calendar day, aligning with typical shift schedules across study ICUs. The dataset was structured in four‐hour intervals, with a single physician and nurse assigned to each interval.

We considered the exposures to be the clinicians, defined as nurse and attending physician assignment during a given interval. Assignment of nurses and physicians to 4‐h intervals was performed using progress notes and meta‐data derived from the EHR. Nurses were identified based on sign‐in logs and physicians were identified based on progress notes that indicated clinical evaluation and management at the time of the interval. All clinician assignment procedures were validated by chart review; more details regarding clinician assignment can be found in the Supporting Information. To ensure model stability, clinicians were excluded if they had < 10 assigned patient encounters over the entire study period.

Acute disease severity was measured using the Laboratory Acute Physiology Score Version 2 (LAPS2) which ranges between 0 (low inpatient mortality risk) and 256 (high inpatient mortality risk). Patients with LAPS2 scores at the time of hospital admission ≥ 50 have clinically significant ICU admission rates, and ≥ 100 have a high risk of mortality or prolonged hospital length of stay [16, 17]. For this study, LAPS2 was calculated for each day that a patient was in the ICU by abstracting vital sign and laboratory values from EHR data that corresponded to the 24 h preceding each day, starting at 7 am of the day of assignment [18]. The most extreme laboratory and vital sign values observed in the preceding 24 h were included in calculations [19, 20, 21]. Our primary outcome was change in LAPS2 from the first day to the last day of ICU admission (Δ*LAPS*). Potential confounding variables included patient age, sex, admission Elixhauser comorbidity index score, location prior to hospital admission (defined as emergency department, direct admission, or outside hospital transfer), medical versus surgical admission (determined using Healthcare Cost and Utilization Project classifications for diagnostic or procedure codes based on the International Classification for Disease, 10th Revision that were associated with encounters), hospital duration prior to ICU admission, interval day of the week, whether the 4‐h interval occurred during the day (7 am–6:59 pm) or night shift (7 pm–6:59 am), and LAPS2 at hospital admission.

### 2.3 Statistical Analyzes

Statistical significance for all two‐sided hypothesis tests was considered at *p* < 0.05. Analyzes were performed using the R programming language (R Foundation for Statistical Computing, Vienna, Austria) and Stata 18 (StataCorp, College Station, Texas, USA).

#### 2.3.1 Model Specification and Performance Assessment

To assess the consistency of clinician value‐add across different patients, and to reduce known risks of VAM that include circularity and model overfitting [13] we performed a random 1:1 split of all patient encounters to create development and test partitions. Using the development partition, we used multivariable linear regression to develop four models: (1) the base model, which regressed Δ*LAPS* onto the previously stated potential confounders; (2) the nurse model, which added interval‐level nurse assignments as a fixed categorical variable to the base model; (3) the physician model, which added interval‐level physician assignments as a fixed categorical variable to the base model; and (4) the combined model, which added both the nurse and physician assignment variables to the base model. To confirm that the clinician assignment variables improved performance of the regression models, we assessed goodness‐of‐fit of each model in the development partition using the coefficient of determination (*R* ^*2*^), the adjusted *R* ^*2*^, and the Akaike information criterion (AIC). We assess relative relationships between clinician roles and Δ*LAPS* based on the difference in the adjusted *R* ^*2*^ between nurses and physicians.

#### 2.3.2 Value‐Add Determination, Performance Definition, and Cross‐Partition Validation

We then implemented the combined model in the testing dataset. Using combined models implemented in both partitions, we extracted the predicted beta coefficients of each individual clinician and, to preserve intuitive interpretations between high value‐add clinicians being associated with more negative (and thus more favorable) Δ*LAPS*, these values were multiplied by negative one.

Based on our modeling approach, the derived clinician value‐add represents a clinician's associated Δ*LAPS* for the average patient that the clinician was assigned to, relative to an arbitrary nurse or physician; it is also specific to the encounter population of a given partition. To demonstrate absolute independent relationships between clinician value‐add and Δ*LAPS*, we fit a fifth linear model that regressed Δ*LAPS* onto the nurse and physician value‐add variables, along with patient age, sex, Elixhauser score, admission source, medical or surgical admission, hospital duration prior to MV initiation, interval day of week, day or night shift of service, admission LAPS, and study ICU. We defined clinician performance as each clinician's marginal associations with Δ*LAPS*. To cross‐validate clinician performance across partitions, we plotted individual clinician performance across both partitions and compared them using the Pearson correlation coefficient (*r*). We interpreted positive correlation as evidence of reproducible individual clinician performance across a variety of clinician encounters.

#### 2.3.3 Evaluation of Random Assignment of Clinicians

Random assignment of clinicians to patients based on their performance is a requirement of VAM and helps mitigate biases from inferential analyzes [13, 22]. We used two approaches to assess the randomness of clinician assignment with respect to performance. First, we used distributional assessments to evaluate relationships between validation partition‐derived clinician performance and patient LAPS2 at the time of first clinician assignment during a patient's encounter in the testing set. Next, we assessed the statistical significance of clinician value‐add terms using linear regression models of initial LAPS2 scores at the beginning of MV initiation in ICUs. Details of both modeling strategies can be found in the Supporting Information.

### 2.4 Sensitivity Analyzes

#### 2.4.1 Missingness Assessment

In the primary analysis, we included all intervals with discernible clinician assignments. In this sensitivity analysis, all missing clinician assignments were instead designated as a single nurse or physician “unknown” assignment in the fixed categorical variable representing clinician assignment. We then assessed model fit of base, physician, nurse, and combined models, as well as the cross‐partition correlation of clinician performance using a 1:1 random sample of patients into development and testing partitions to confirm robustness of our performance determination approach to inclusion of the missing assignments.

#### 2.4.2 Temporality Assessment

Patients who experience an ICU length of stay later than 7 days may make a transition to chronic critical illness, in which acute disease severity scores may no longer accurately predict long term outcomes [23]. To assess cross‐partition consistency of value‐add estimates that are based on disease severity trajectories that occurred early during the mechanical ventilation period, we performed the above cross‐partition validation using assignments and Δ*LAPS* that occurred within the first 7 days of mechanical ventilation. This analysis formally omitted any clinician‐encounter assignments that occurred 7 days after the time of mechanical ventilation initiation and assessed cross partition correlation (*r*) of the marginal predicted Δ*LAPS* for each individual clinician stratified by clinician role.

## 3 Results

We identified 15,520 mechanically ventilated patients with qualifying clinician assignments between 2018 and 2022, accounting for 17,082 unique ICU encounters and 768,786 4‐h intervals. We identified 1719 nurses and 215 physicians who were each assigned across at least 10 distinct clinical encounters. Among all intervals, nurse assignments were missing for 39,392 (4.3%) and physician assignments were missing for 68,671 (7.9%). After randomly partitioning ICU encounters, the complete case primary analysis included a development partition with 8556 encounters among 8133 patients, and a test partition with 8526 encounters among 8029 patients (Table 1). Distributions of study covariates were similar across partitions.

**TABLE 1:** Baseline covariates of study population.

	All^a^ *n* = 15,520	Development^a^ *n* = 8133	Test^a^ *n* = 8029
Change in LAPS2 during MV duration, median (IQR)	−22 (−55, 4)	−22 (−54, 3)	−21 (−55, 4)
Elixhauser comorbidity index score, median (IQR)	19 (6, 33)	19 (6, 33)	19 (6, 33)
LAPS2 at admission, mean (SD)	143 (51)	143 (50)	143 (51)
Age, median (IQR)	63 (51, 72)	63 (51, 72)	62 (51, 72)
Hospital duration prior to eligibility (hours), median (IQR)	11 (4, 76)	11 (4, 75)	11 (4, 76)
Hospital length of stay, median hours (IQR)	349 (190, 641)	354 (194, 642)	344 (187, 640)
ICU length of stay, median hours (IQR)	135 (70, 281)	137 (71, 277)	133 (70, 284)
Mechanical ventilation duration, median hours (IQR)	61 (25, 159)	62 (25, 159)	60 (25, 159)
Duration of time eligible for study inclusion, median hours (IQR)	122 (67, 261)	124 (67, 260)	120 (67, 262)
Number of intervals, median (IQR)	89.0 (81.0, 105.0)	88.2 (78.0, 104.0)	89.9 (83.0, 106.0)
Proportion of shifts that occurred during daytime, median (IQR)	0.5 (0.5, 1.0)	0.5 (0.5, 1.0)	0.5 (0.5, 1.0)
Number of nurses per encounter, median (IQR)	17.3 (16.0, 16.0)	17.1 (16.0, 16.0)	17.5 (17.0, 16.0)
Number of shifts per nurse, median (IQR)	3.0 (3.0; 0.0)	3.0 (3.0, 0.0)	3.0 (3.0, 0.0)
Number of physicians per encounter, median (IQR)	3.5 (3.0, 3.0)	3.4 (3.0, 3.0)	3.5 (3.0, 3.0)
Number of intervals per physician, median (IQR)	5.9 (6.0, 0.0)	5.9 (6.0, 0.0)	5.9 (6.0, 0.0)
Medical versus surgical admissions			
Medical admissions, *n* (%)	7776 (44.4%)	3860 (44.1%)	3916 (44.8%)
Surgical admissions, *n* (%)	9711 (55.5%)	4883 (55.8%)	4828 (55.2%)
Missing admission type, *n* (%)	8 (< 1%)	5 (0.1%)	3 (< 1%)
Female sex	7210 (41.2%)	3626 (41.4%)	3584 (41.0%)
Admission source			
ED admissions, *n* (%)	7405 (42.4%)	3684 (42.2%)	3721 (42.6%)
Direct admissions, *n* (%)	3868 (22.1%)	1955 (22.4%)	1913 (21.9%)
Outside facility transfers, *n* (%)	6202 (35.5%)	3099 (35.5%)	3103 (35.5%)

^a^ Mean (Median; IQR); *n* (%).

### 3.1 Model Performance

The base model yielded an adjusted *R* ^2^ of 0.12 and AIC of 3,980,745. Model fit was improved by adding nurse (adjusted *R* ^2^ 0.18, AIC 3,959,350) and physician (adjusted *R* ^2^ 0.16, AIC 3,963,114) assignments. The combined model had best model fit with an adjusted *R* ^2^ of 0.19 and AIC of 3,953,236 (Table 2). The improvement in adjusted *R* ^2^ across the models indicated that, combined, nurse and physician assignments accounted for 7% of variability in Δ*LAPS*. Independently, nurse assignments accounted for greater variability in Δ*LAPS* compared to physician assignments (6% vs. 4%, respectively).

**TABLE 2:** Model performance.

	Base model	Nurse model	Physician model	Combined model
*R* ^2^	0.12	0.18	0.16	0.19
Adjusted *R* ^2^	0.12	0.18	0.16	0.19
AIC	3,980,745	3,959,350	3,963,114	3,953,236

*Note:* Base models were adjusted for patient age, sex, admission Elixhauser comorbidity index score, admission source, medical versus surgical admission, hospital duration prior to ICU admission, day of week, LAPS2 at ICU admission, and LAPS2 on day of service. The physician model contained the same variables as the base model and was additionally adjusted for physician assignments. The nurse model contained the same variables as the base model and was additionally adjusted for nursing assignments. The combined model contained the same variables as the base model and was additionally adjusted for both physician and nurse assignments.

Abbreviations: AIC—Akaike information criterion, *R* ^2^—Coefficient of determination.

### 3.2 Value‐Add Determination and Performance Correlation Across Partitions

**FIGURE 1:**
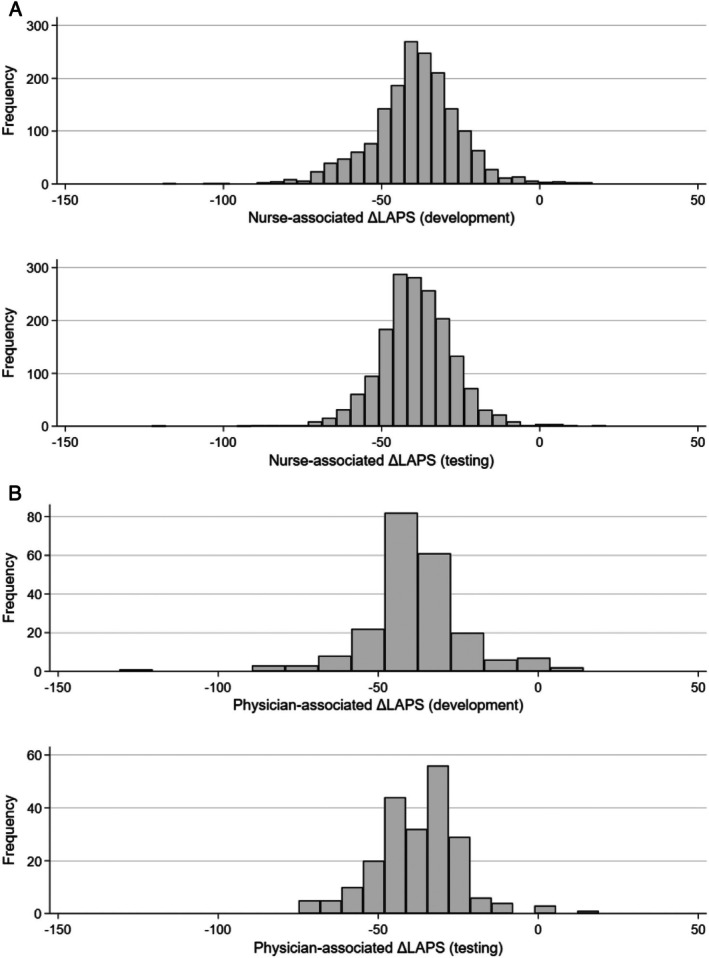
(A) Frequency distribution of nurse associated change in disease severity during intensive care unit admission. Top panel: Frequency distribution in development partition. Bottom panel: Frequency distribution in testing partition. Δ*LAPS*‐change in Laboratory Acute Physiology Score 2 from the beginning to end of intensive care unit admission. (B) Frequency distribution of physician associated change in disease severity during intensive care unit admission. Top panel: Frequency distribution in development partition. Bottom panel: Frequency distribution in testing partition. Δ*LAPS*‐change in Laboratory Acute Physiology Score 2 from the beginning to end of intensive care unit admission.

We used coefficients derived from the combined model in the development partition to define nurse and physician value‐add; for both clinician types, increased value‐add was associated with a larger magnitude of Δ*LAPS* decrease. The smallest observed nurse value‐add was associated with a +17‐point increase in LAPS2 (95% CI: [12, 22], *p* < 0.001) and the largest with a −119‐point decrease (95% CI: [−126, −112], *p* < 0.001). The smallest observed physician value‐add was associated with a +14‐point increase in LAPS2 (95% CI: [6, 22], *p* < 0.001) and the largest was associated a −131‐point decrease (95% CI: [−144, −118], *p* < 0.001; Figure 1, Table S1). Using coefficients derived from the combined model, measures of performance in the development and the testing partitions were found to be positively correlated (nurses *r* = 0.218, *p* < 0.001; physicians *r* = 0.322, *p* < 0.001; Figures S1 and S2).

### 3.3 Evaluation of VAM Model Assumption: Random Assignment

Clinician performance was not associated with initial LAPS2 at time of clinician assignment (Figure S3), and we identified no significant relationships between LAPS2 at the time of mechanical ventilation and nurse (*p* = 0.091) or physician value‐add (*p* = 0.523, Table S2).

### 3.4 Sensitivity Analyzes

In the missingness sensitivity analysis, each missing clinician assignment was encoded as an unknown physician or nurse respectively, leading to a final dataset that contained 870,383 intervals across 17,495 ICU admissions and 15,887 patients. Baseline characteristics were similar to the primary analysis (Table S3). Consistent with the primary analysis, combined models remained superior to base, nurse, and physician models (Table S4). Performance was approximately normally distributed (Figure S4) with correlation between the development and test partitions comparable to the primary analysis (Figure S5). Clinician performance was not meaningfully associated with patient acute disease severity at the time of initial assignment in the sensitivity analysis (Figure S6).

In the temporality sensitivity analysis, marginal associated clinician Δ*LAPS* was of smaller magnitude compared to the primary analysis (nurse median [IQR]: −16 [−22, −9] for development partition, −16 [−24, −9] for testing partition; physician median [IQR]: −16 [−22, −8] for development partition, −17 [−22, −8] for testing partition, Figure S7). Cross‐partition correlation was poor for nurses (*r* = 0.046, *p* = 0.140) and moderate for physicians (*r* = 0.373, *p* < 0.001; Figure S8).

## 4 Discussion

In a retrospective cohort of mechanically ventilated patients, we quantified independent nurse and physician performance for reducing patients' illness severity in an interprofessional team‐based care model. Together, nurse and physician value‐add were associated with 7% of variability in Δ*LAPS* in this cohort. Our findings are consistent with a prior study in which nurse value‐add was associated with 8% of disease severity variability [13]. Relative to the base model containing patient‐level characteristics expected to influence illness trajectory, clinician assignments increased the explained variance in Δ*LAPS* by almost 60%. In addition, nurse assignments in mechanical ventilation may be more associated with disease severity variability, because the two‐clinician model only added a single percentage point of variability explained to the nested nurse‐only model. Since model fit improved with workers across two disciplines, clinical VAMs are likely to be optimized when many interprofessional roles can be included in the modeling strategy. Additionally, models built on clinician care throughout ICU admission resulted in more consistent nurse performance estimates, which aligns with existing evidence that outcomes of mechanical ventilation patients depends on clinical care delivered even in the post extubation period [24, 25, 26, 27].

Quantifying clinician value‐add facilitates numerous opportunities for clinical outcome assessment, intervention development, and quality improvement [28]. Because our study demonstrates that individual clinicians have independent relationships with disease severity changes across patients, and because these relationships vary between clinicians, clinician value‐add may have relevance for effective resource administration and ideal interdisciplinary team structuring.

This study has limitations. First, though missingness was relatively low for physician and nurse assignments and our findings demonstrated robustness to missingness, our results may be biased if missingness occurred not at random. Second, challenges in clear identification of workers from other disciplines, such as respiratory therapists, precluded their inclusion in this study. Finally, unmeasured confounding is a potential threat of any retrospective analysis, particularly for VAM. Specifically, there may be multiple structural, institutional, and patient‐level factors beyond those captured in our analysis that might influence clinician performance. Value‐add estimates might be therefore influenced by a range of factors including hospital resource availability, clinical care processes and routine service lines, clinician expertise, and even cognitive biases. For these reasons, we caution against value‐add determinations as components of quality assessments per se. However, future work might assess whether other hospital and patient‐related factors may themselves be associated with variability in clinician value‐add.

This study also has strengths. This is the first to adapt VAM to an interprofessional healthcare team context. Other advantages include the large population size, sociodemographic diversity of the study population, and employment of detailed patient‐level EHR data. Lastly, because our determination of clinician value‐add satisfies VAM assumptions, this approach gives some confidence that our results are less prone to bias and satisfies a requirement for future causal inference analyzes [22].

## 5 Conclusion

Clinician value‐add can be determined for nurses and physicians who care for mechanically ventilated patients during interprofessional team‐based care. Nurses and physicians are independently and jointly associated with disease severity trajectory. Because clinician performance is independently associated with a wide range of disease severity changes, future studies should further explore relationships between clinician performance and clinical outcomes.

## Author Contributions

**Meeta Kerlin**, **Christopher F. Chesley**, **Rachel Kohn**, **Hayley B. Gershengorn**, **Kelly C. Vranas**, **Catherine L. Hough**, **Olga Yakusheva**, **Deena Kelly Costa**, **Deanna J. Marriott**, and **David A. Asch:** study conception and design. **Stefania Scott:** data collection. **Christopher F. Chesley**, **Yingying Lu**, and **Wei Wang:** data analysis. **Christopher F. Chesley** and **Meeta Kerlin:** writing of manuscript. Manuscript revisions for content and approval: All authors.

## Funding

C.F.C. is funded by NHLBI K01HL171466. C.F.C. and M.K. were funded by NHLBI R01HL146386. HBG receives funding from the University of Miami Hospital and Clinics Data Analytics Research Team (UHealth‐DART).

## Conflicts of Interest

The authors declare no conflicts of interest.

## Supporting information


**Figure S1:** Correlation of nurse‐associated Δ*LAPS* between development and test partitions. These graphs plot the predicted clinician value using the combined model across and paired between the development and test partitions. E1. Paired predicted nurse value. E2. Paired predicted physician value. For both correlation coefficients, *p* < 0.001.

**Figure S2:** Correlation of clinician value between development and test sets. These graphs plot the predicted clinician value using the combined model across and paired between the development and test sets. E1. Paired predicted nurse value. E2. Paired predicted physician value. For both correlation coefficients, *p* < 0.001.

**Figure S3A:** Distribution of nurse‐associated LAPS2 score at the time of first nurse assignment. LAPS2‐ Laboratory acute physiology score 2; Δ*LAPS*‐change in Laboratory Acute Physiology Score 2 from the beginning to end of intensive care unit admission.

**Figure S3B:** Distribution of physician‐associated LAPS2 score at the time of first physician assignment. LAPS2‐ Laboratory acute physiology score 2; Δ*LAPS*‐change in Laboratory Acute Physiology Score 2 from the beginning to end of intensive care unit admission.

**Figure S4A:** Frequency distribution of nurse associated change in disease severity during intensive care unit admission in the missingness sensitivity analysis. Top panel: frequency distribution in development partition. Bottom panel: frequency distribution in testing partition. Δ*LAPS*‐change in Laboratory Acute Physiology Score 2 from the beginning to end of intensive care unit admission.

**Figure S4B:** Frequency distribution of physician associated change in disease severity during intensive care unit admission in the missingness sensitivity analysis. Top panel: frequency distribution in development partition. Bottom panel: frequency distribution in testing partition. Δ*LAPS*‐change in Laboratory Acute Physiology Score 2 from the beginning to end of intensive care unit admission.

**Figure S5A:** Correlation of nurse‐associated Δ*LAPS* between development and test partitions in the missingness sensitivity analysis. These graphs plot the predicted clinician value using the combined model across and paired between the development and test partitions. 2A. Paired predicted nurse value. 2B. Paired predicted physician value. For both correlation coefficients, *p* < 0.001.

**Figure S5B:** Correlation of clinician value between development and test sets in the missingness sensitivity analysis. These graphs plot the predicted clinician value using the combined model across and paired between the development and test sets. 2A. Paired predicted nurse value. 2B. Paired predicted physician value. For both correlation coefficients, *p* < 0.001.

**Figure S6A:** Distribution of nurse‐associated LAPS2 score at the time of first nurse assignment in the missingness sensitivity analysis. LAPS2‐ Laboratory acute physiology score 2; Δ*LAPS*‐change in Laboratory Acute Physiology Score 2 from the beginning to end of intensive care unit admission.

**Figure S6B:** Distribution of physician‐associated LAPS2 score at the time of first physician assignment in the missingness sensitivity analysis. LAPS2‐ Laboratory acute physiology score 2; Δ*LAPS*‐change in Laboratory Acute Physiology Score 2 from the beginning to end of intensive care unit admission.

**Figure S7A:** Frequency distribution of nurse associated change in disease severity during intensive care unit admission in the first 7 days of mechanical ventilation. Top panel: frequency distribution in development partition. Bottom panel: frequency distribution in testing partition. Δ*LAPS*‐change in Laboratory Acute Physiology Score 2 from the beginning to end of intensive care unit admission.

**Figure S7B:** Frequency distribution of physician associated change in disease severity during intensive care unit admission in the first 7 days of mechanical ventilation. Top panel: frequency distribution in development partition. Bottom panel: frequency distribution in testing partition. Δ*LAPS*‐change in Laboratory Acute Physiology Score 2 from the beginning to end of intensive care unit admission.

**Figure S8A:** Correlation of nurse‐associated Δ*LAPS* between development and test partitions in the first 7 days of mechanical ventilation. These graphs plot the predicted clinician value using the combined model across and paired between the development and test partitions. 8A. Paired predicted nurse value. 8B. Paired predicted physician value. For both correlation coefficients, *p* < 0.001.

**Figure S8B:** Correlation of nurse‐associated Δ*LAPS* between development and test partitions in the first 7 days of mechanical ventilation. These graphs plot the predicted clinician value using the combined model across and paired between the development and test partitions. 8A. Paired predicted nurse value. 8B. Paired predicted physician value. For both correlation coefficients, *p* < 0.001.

**Table S1:** Summary statistics for marginal change in Laboratory Acute Physiology Score 2 based on combined models.
**Table S2:** Clinician value‐add relationships with disease severity at mechanical ventilation initiation. Presented are model coefficients from a linear regression testing independent relationships between clinician value‐add and disease severity at mechanical ventilation initiation as measured by the Laboratory Acute Physiology Score 2. CI‐ confidence interval; ED‐ emergency department; ICU‐ intensive care unit; LAPS2‐ Laboratory Acute Physiology Score 2; MV‐ mechanical ventilation; NVA‐ nurse value‐add; PVA‐ physician value‐add; Ref‐ reference variable.
**Table S3:** Baseline characteristics of imputation dataset. ^1^Mean (Median; IQR); *n* (%).
**Table S4:** Imputed model performance. All models were built using the imputation approach described in the text. Base models were adjusted for patient age, gender, admission Elixhauser score, admission source, medical versus surgical admission, hospital duration prior to ICU admission, day of week, LAPS2 at ICU admission, and LAPS2 on day of service. Physician model contained the same variables as the base model and was additionally adjusted for physician assignments. Nurse model contained the same variables as the base model and was additionally adjusted for physician assignments. The combined model contained the same variables as the base model and was additionally adjusted for both physician and nurse assignments. AIC‐ Akaike information criterion, *R* ^2^‐ coefficient of determination.

## Data Availability

Research data are not shareable due to human subject protections.
