# Supplementary figures and images for: Quantifying Nurse and Physician Clinical Performance for Mechanically Ventilated Patients
Source: Health Serv Res. 2026 Jul 29;61(4):e70153. doi: 10.1111/1475-6773.70153 (PMC13417481; doi:10.1111/1475-6773.70153)

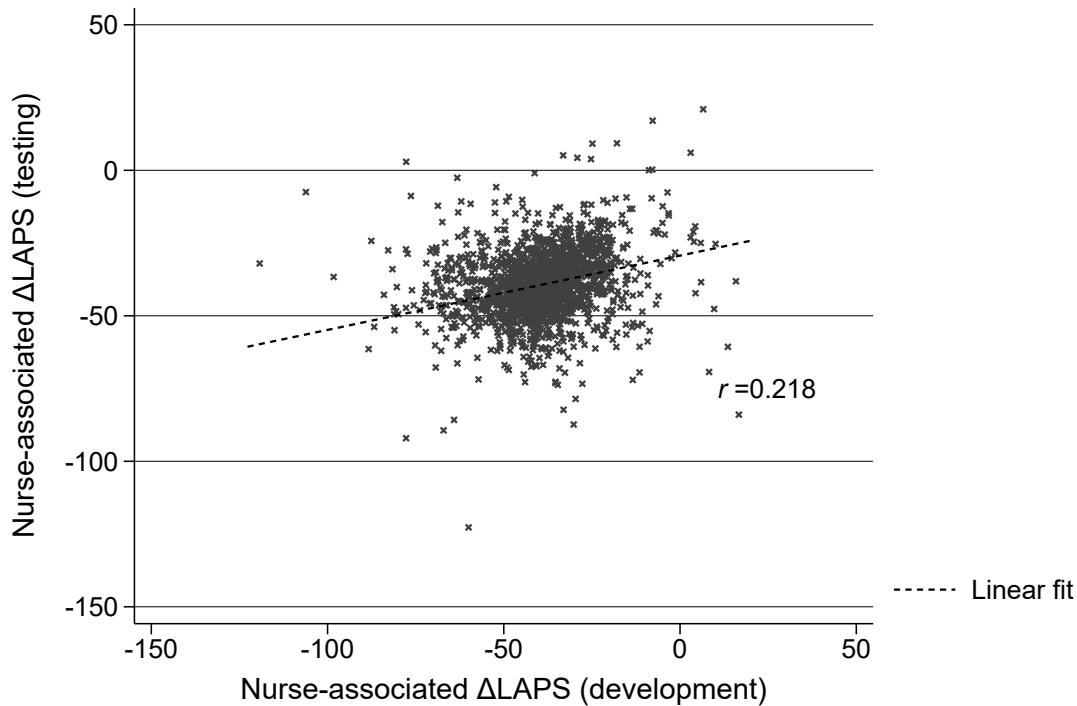

Supplement: Supplementary file 1 — Figure S1: Correlation of nurse‐associated ΔLAPS between development and test partitions. These graphs plot the predicted clinician value using the combined model across and paired between the development and test partitions. E1. Paired predicted nurse value. E2. Paired predicted physician value. For both correlation coefficients, p < 0.001. [file HESR-61-e70153-s010.pdf]

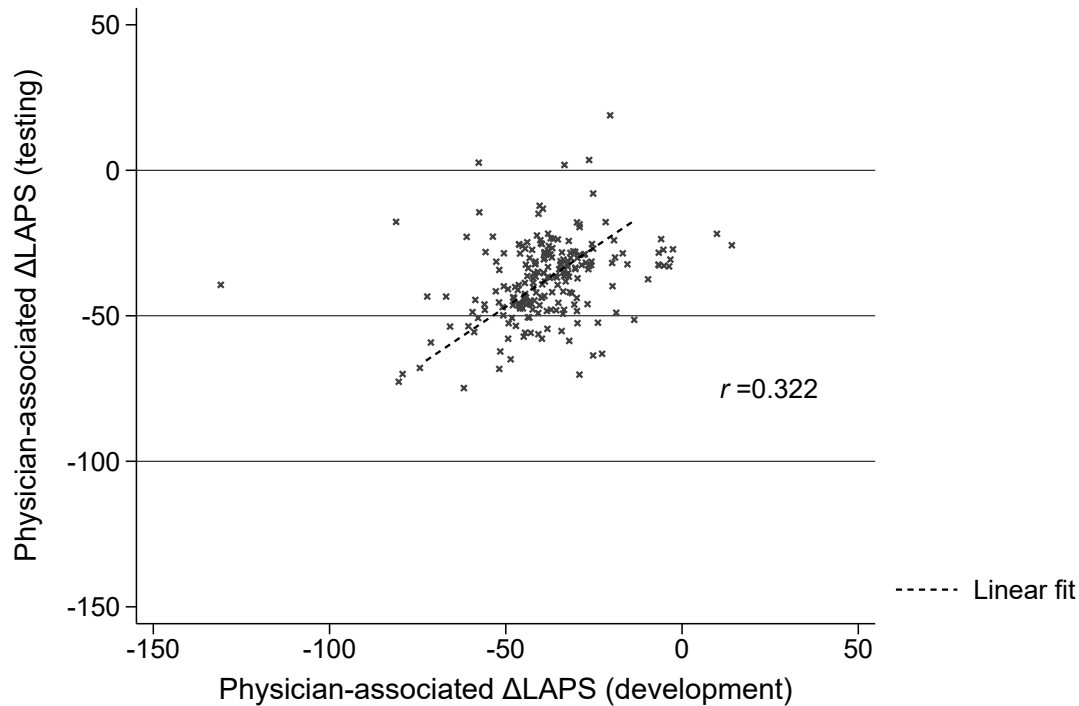

Supplement: Supplementary file 2 — Figure S2: Correlation of clinician value between development and test sets. These graphs plot the predicted clinician value using the combined model across and paired between the development and test sets. E1. Paired predicted nurse value. E2. Paired predicted physician value. For both correlation coefficients, p < 0.001. [file HESR-61-e70153-s006.pdf]

Nurse-associated  $\Delta$ LAPS

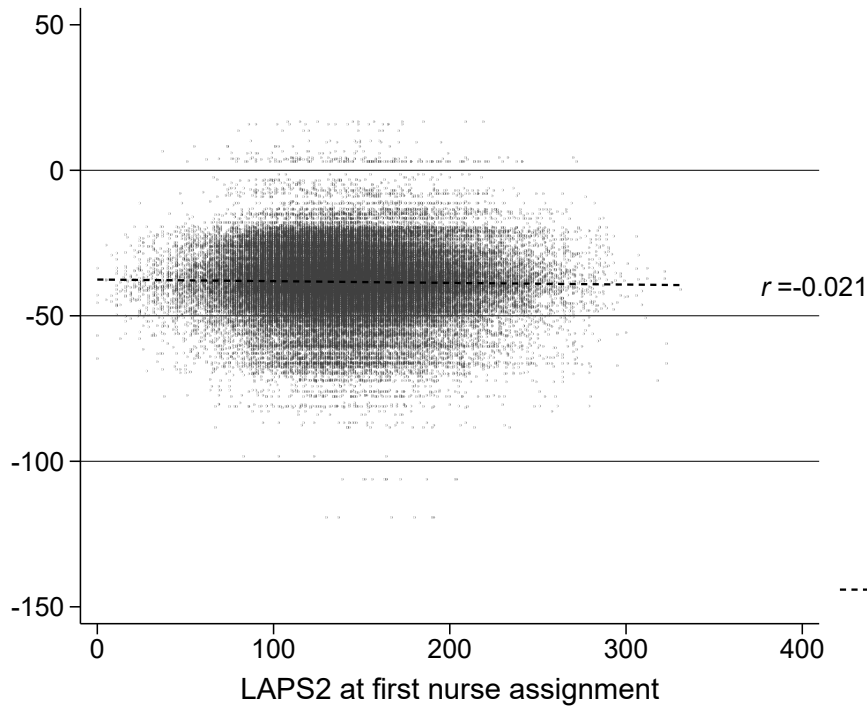

Supplement: Supplementary file 3 — Figure S3A: Distribution of nurse‐associated LAPS2 score at the time of first nurse assignment. LAPS2‐ Laboratory acute physiology score 2; ΔLAPS‐change in Laboratory Acute Physiology Score 2 from the beginning to end of intensive care unit admission. [file HESR-61-e70153-s013.pdf]

Physician-associated  $\Delta$ LAPS

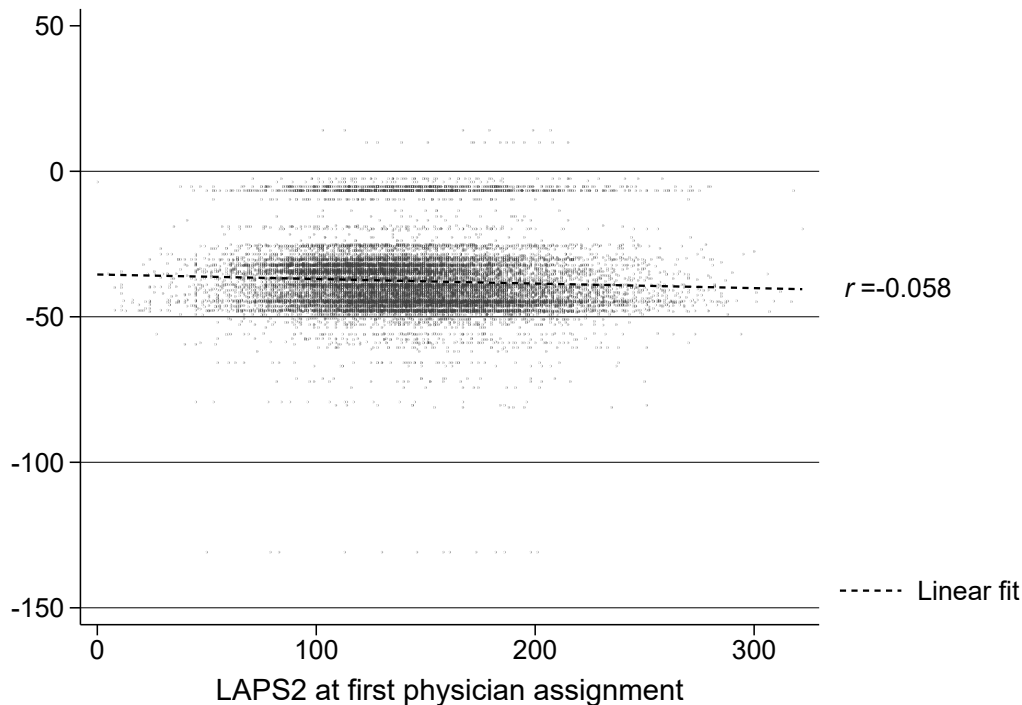

Supplement: Supplementary file 4 — Figure S3B: Distribution of physician‐associated LAPS2 score at the time of first physician assignment. LAPS2‐ Laboratory acute physiology score 2; ΔLAPS‐change in Laboratory Acute Physiology Score 2 from the beginning to end of intensive care unit admission. [file HESR-61-e70153-s004.pdf]

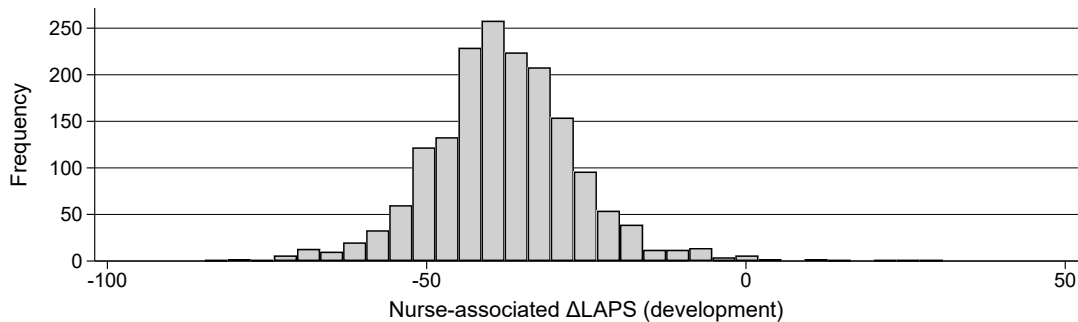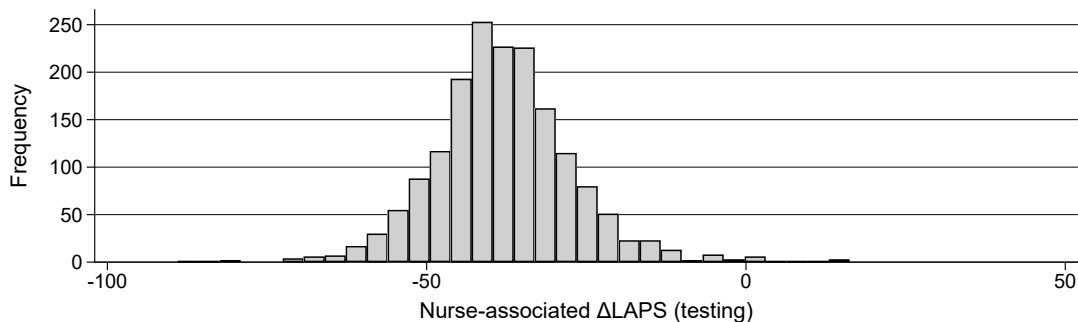

Supplement: Supplementary file 5 — Figure S4A: Frequency distribution of nurse associated change in disease severity during intensive care unit admission in the missingness sensitivity analysis. Top panel: frequency distribution in development partition. Bottom panel: frequency distribution in testing partition. ΔLAPS‐change in Laboratory Acute Physiology Score 2 from the beginning to end of intensive care unit admission. [file HESR-61-e70153-s014.pdf]

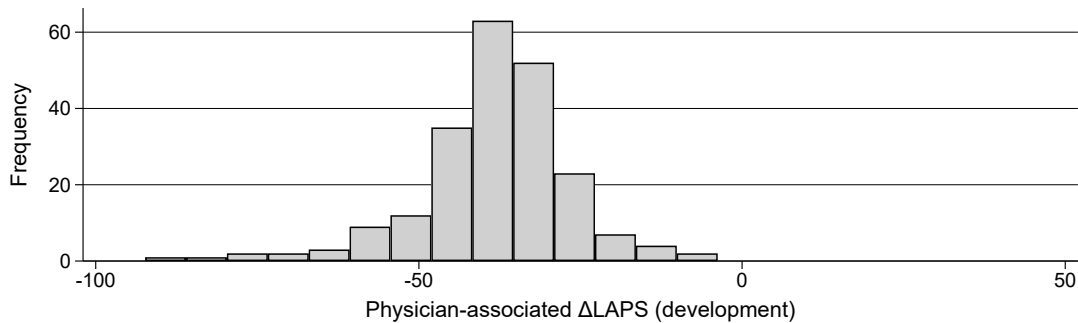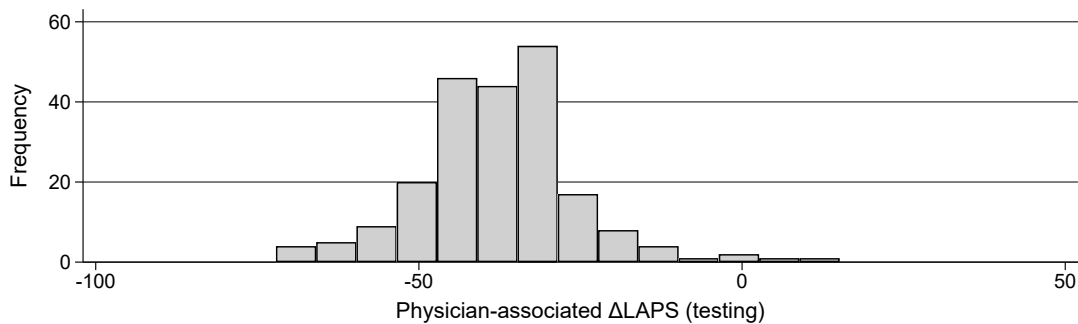

Supplement: Supplementary file 6 — Figure S4B: Frequency distribution of physician associated change in disease severity during intensive care unit admission in the missingness sensitivity analysis. Top panel: frequency distribution in development partition. Bottom panel: frequency distribution in testing partition. ΔLAPS‐change in Laboratory Acute Physiology Score 2 from the beginning to end of intensive care unit admission. [file HESR-61-e70153-s011.pdf]

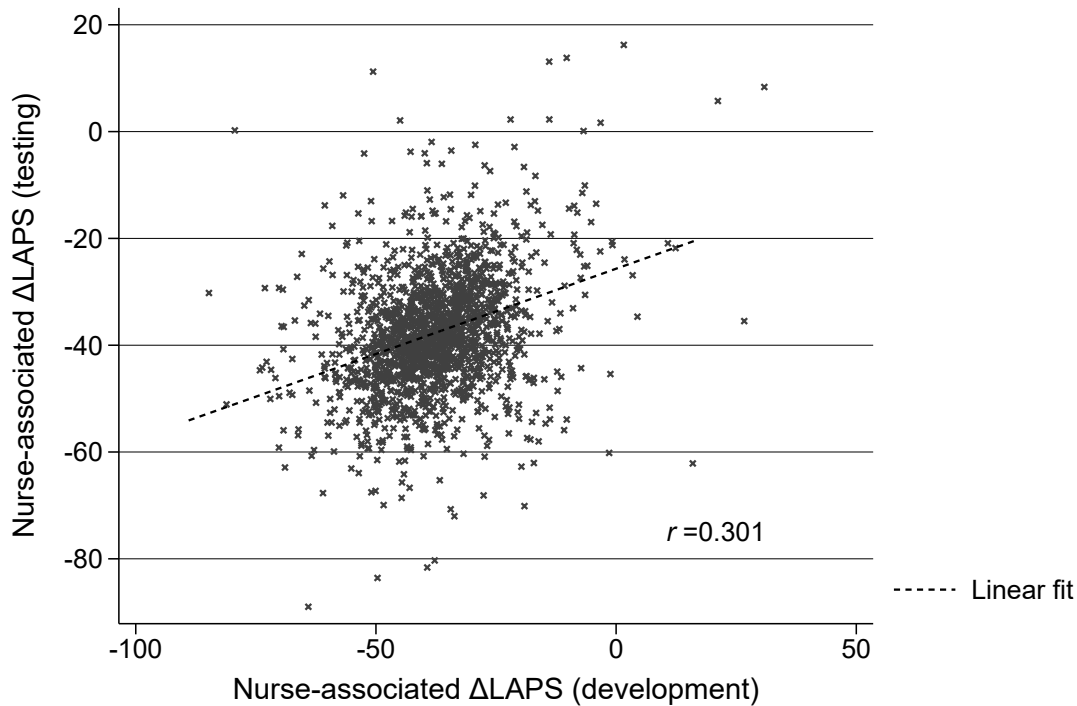

Supplement: Supplementary file 7 — Figure S5A: Correlation of nurse‐associated ΔLAPS between development and test partitions in the missingness sensitivity analysis. These graphs plot the predicted clinician value using the combined model across and paired between the development and test partitions. 2A. Paired predicted nurse value. 2B. Paired predicted physician value. For both correlation coefficients, p < 0.001. [file HESR-61-e70153-s007.pdf]

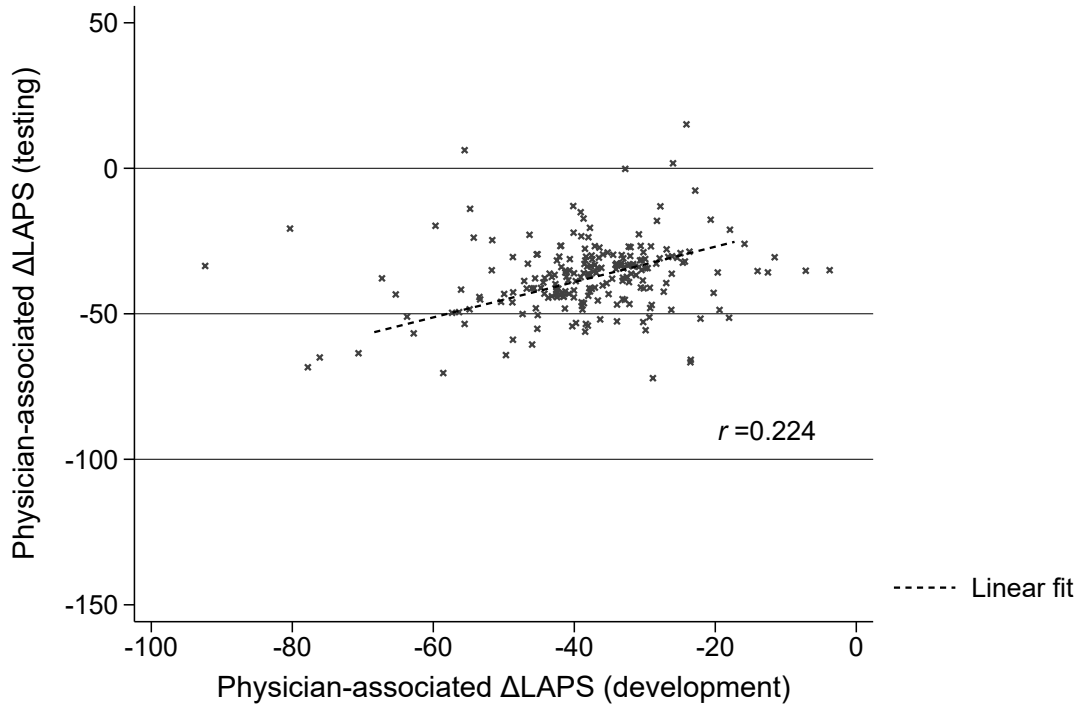

Supplement: Supplementary file 8 — Figure S5B: Correlation of clinician value between development and test sets in the missingness sensitivity analysis. These graphs plot the predicted clinician value using the combined model across and paired between the development and test sets. 2A. Paired predicted nurse value. 2B. Paired predicted physician value. For both correlation coefficients, p < 0.001. [file HESR-61-e70153-s008.pdf]

Nurse-associated  $\Delta$ LAPS

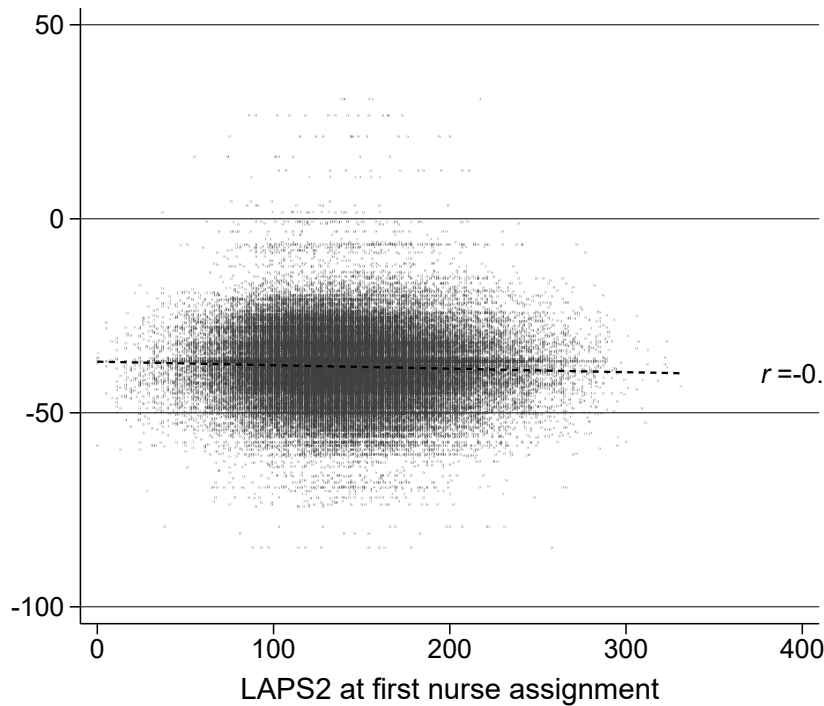

Supplement: Supplementary file 9 — Figure S6A: Distribution of nurse‐associated LAPS2 score at the time of first nurse assignment in the missingness sensitivity analysis. LAPS2‐ Laboratory acute physiology score 2; ΔLAPS‐change in Laboratory Acute Physiology Score 2 from the beginning to end of intensive care unit admission. [file HESR-61-e70153-s015.pdf]

Physician-associated  $\Delta$ LAPS

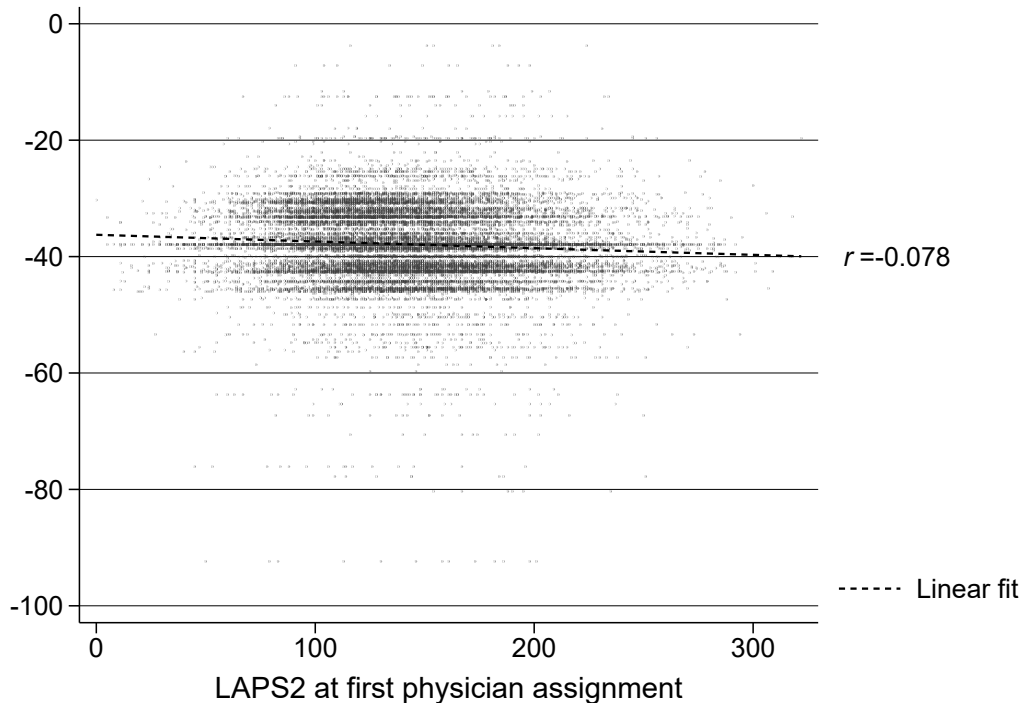

Supplement: Supplementary file 10 — Figure S6B: Distribution of physician‐associated LAPS2 score at the time of first physician assignment in the missingness sensitivity analysis. LAPS2‐ Laboratory acute physiology score 2; ΔLAPS‐change in Laboratory Acute Physiology Score 2 from the beginning to end of intensive care unit admission. [file HESR-61-e70153-s005.pdf]

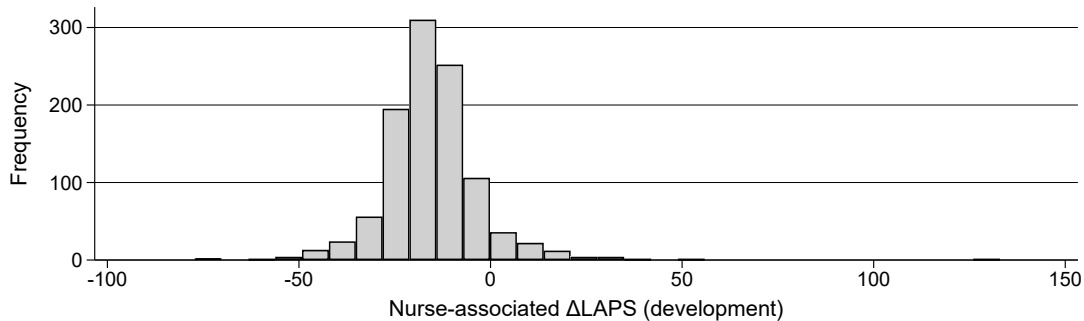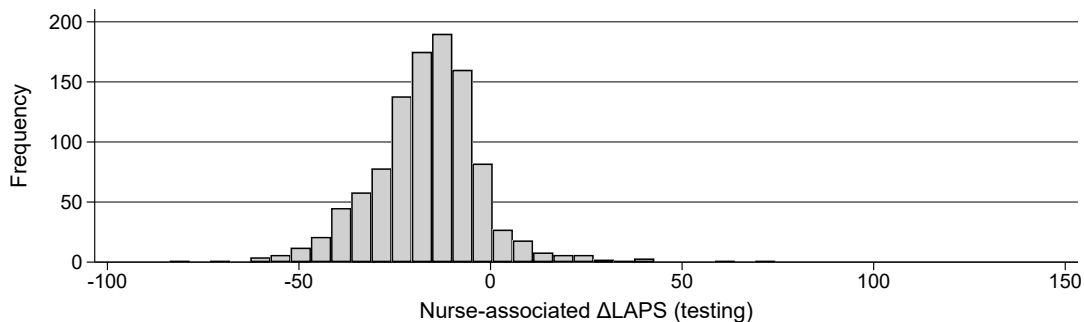

Supplement: Supplementary file 11 — Figure S7A: Frequency distribution of nurse associated change in disease severity during intensive care unit admission in the first 7 days of mechanical ventilation. Top panel: frequency distribution in development partition. Bottom panel: frequency distribution in testing partition. ΔLAPS‐change in Laboratory Acute Physiology Score 2 from the beginning to end of intensive care unit admission. [file HESR-61-e70153-s001.pdf]

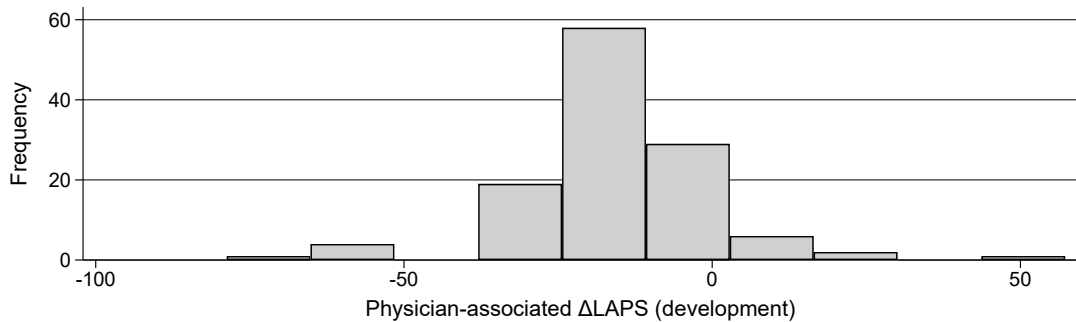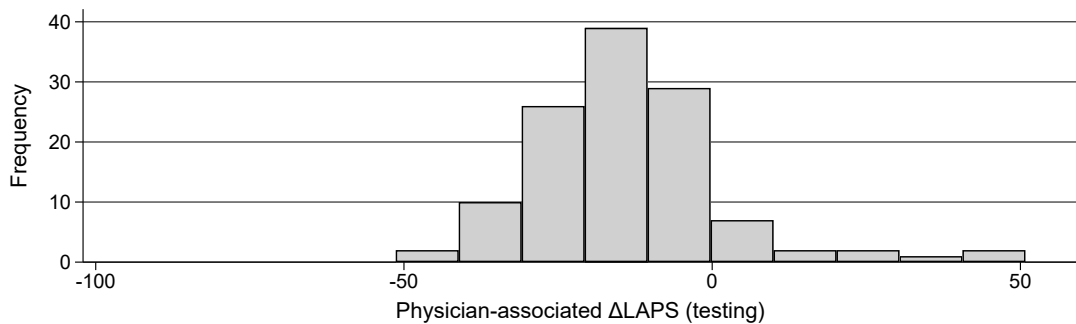

Supplement: Supplementary file 12 — Figure S7B: Frequency distribution of physician associated change in disease severity during intensive care unit admission in the first 7 days of mechanical ventilation. Top panel: frequency distribution in development partition. Bottom panel: frequency distribution in testing partition. ΔLAPS‐change in Laboratory Acute Physiology Score 2 from the beginning to end of intensive care unit admission. [file HESR-61-e70153-s002.pdf]

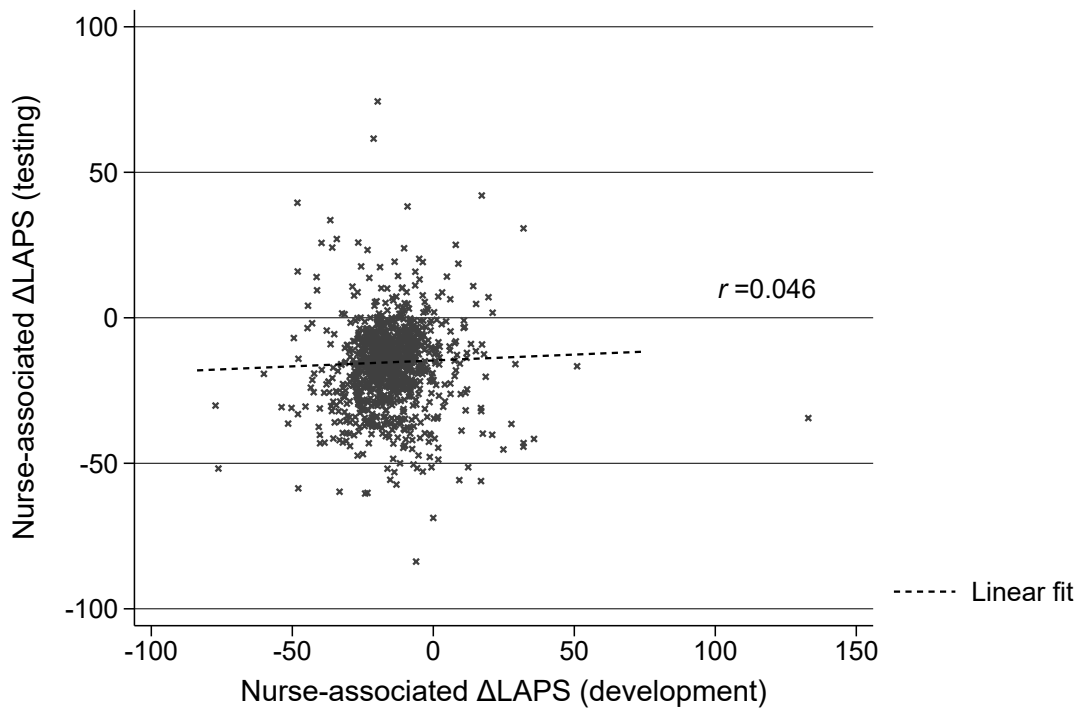

Supplement: Supplementary file 13 — Figure S8A: Correlation of nurse‐associated ΔLAPS between development and test partitions in the first 7 days of mechanical ventilation. These graphs plot the predicted clinician value using the combined model across and paired between the development and test partitions. 8A. Paired predicted nurse value. 8B. Paired predicted physician value. For both correlation coefficients, p < 0.001. [file HESR-61-e70153-s003.pdf]

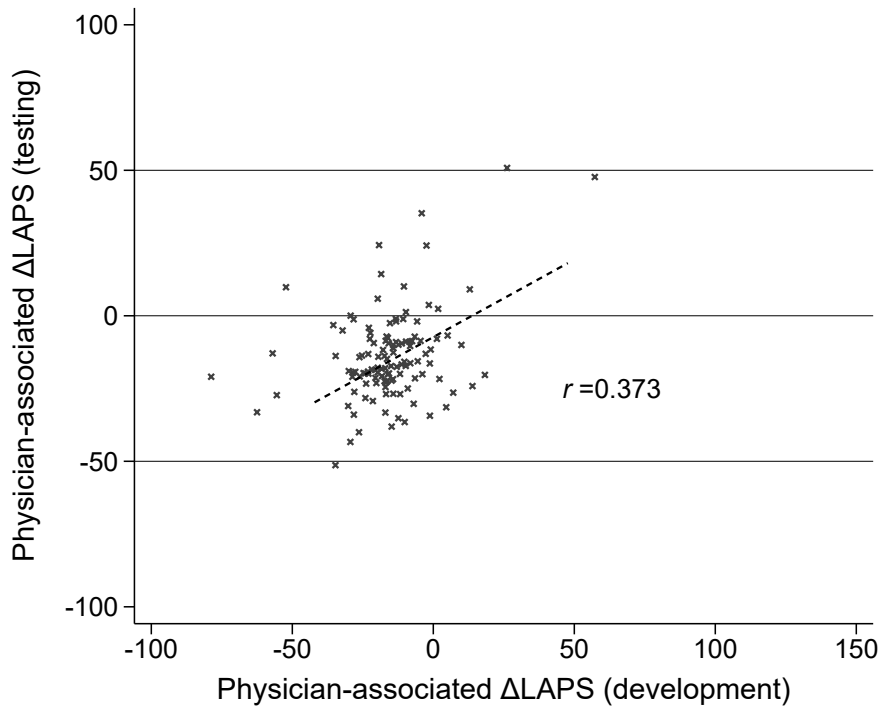

----- Linear fit

Supplement: Supplementary file 14 — Figure S8B: Correlation of nurse‐associated ΔLAPS between development and test partitions in the first 7 days of mechanical ventilation. These graphs plot the predicted clinician value using the combined model across and paired between the development and test partitions. 8A. Paired predicted nurse value. 8B. Paired predicted physician value. For both correlation coefficients, p < 0.001. [file HESR-61-e70153-s012.pdf]
