# Supplementary material for: Quantifying Nurse and Physician Clinical Performance for Mechanically Ventilated Patients
Source: Health Serv Res. 2026 Jul 29;61(4):e70153. doi: 10.1111/1475-6773.70153 (PMC13417481; doi:10.1111/1475-6773.70153)
Supplement: Supplementary file 15 — Table S1: Summary statistics for marginal change in Laboratory Acute Physiology Score 2 based on combined models. Table S2: Clinician value‐add relationships with disease severity at mechanical ventilation initiation. Presented are model coefficients from a linear regression testing independent relationships between clinician value‐add and disease severity at mechanical ventilation initiation as measured by the Laboratory Acute Physiology Score 2. CI‐ confidence interval; ED‐ emergency department; ICU‐ intensive care unit; LAPS2‐ Laboratory Acute Physiology Score 2; MV‐ mechanical ventilation; NVA‐ nurse value‐add; PVA‐ physician value‐add; Ref‐ reference variable. Table S3: Baseline characteristics of imputation dataset. 1Mean (Median; IQR); n (%). Table S4: Imputed model performance. All models were built using the imputation approach described in the text. Base models were adjusted for patient age, gender, admission Elixhauser score, admission source, medical versus surgical admission, hospital duration prior to ICU admission, day of week, LAPS2 at ICU admission, and LAPS2 on day of service. Physician model contained the same variables as the base model and was additionally adjusted for physician assignments. Nurse model contained the same variables as the base model and was additionally adjusted for physician assignments. The combined model contained the same variables as the base model and was additionally adjusted for both physician and nurse assignments. AIC‐ Akaike information criterion, R 2‐ coefficient of determination. [file HESR-61-e70153-s009.docx]

**Title:** Quantifying nurse and physician clinical performance for mechanically ventilated patients

**Online data supplement**

**Supplemental Methods**

Clinician assignments overview

For each encounter, each four-hour interval during the eligibility period (defined as the start of invasive mechanical ventilation [IMV] in the intensive care unit [ICU] until the end of the ICU admission) was assigned one nurse and one physician using electronic health record (EHR) data and meta-data. Two separate algorithms were used for nurses and physicians respectively, detailed below. Algorithms were developed based on investigators’ (MPK, CC, RK) clinical experience with how clinician assignments are captured by the EHR in the health system of study and knowledge of staff scheduling across study ICUs. Clinician assignment rules were iteratively refined by chart review; with each refinement, 10-20 charts were manually reviewed (MPK, CC, AB) to evaluate accuracy of the assignment by the algorithm, using the gold standard of EHR documentation (determined based on EHR signatures for vital sign documentation [by nurses], and written notes [by physicians and nurses]). Once accuracy was estimated to be at least 95%, the algorithm was considered final. If it was impossible to create an assignment based on the steps detailed below, then the assignment was categorized as “unknown.” Unknown assignments mainly occurred due to a nurse not having been signed in for a four-hour interval, or an attending physician note being unavailable.

Nurse assignments

The nurse assignment algorithm utilized data from clinicians’ assignments to patient care roles. Nurses routinely work 8-, 12-, and rarely 24-hour worker shifts in study ICUs. They typically sign in at the beginning and sign out at the end of shifts to indicate their assignment to patients, creating a timestamped EHR signature for the beginning and end of their shifts. To assign nurses to study intervals, we followed a series of rules:

1. If a four-hour interval was entirely included within a nurse shift – that is, the start time of the interval was after the start time of the nurse shift and the end time of the interval was before the end of the nurse shift – then that nurse was assigned to that interval.
2. Because signing in and out was a manual process, nurses sometimes sign in slightly before or after the start of a shift and may sign out slightly before or after the end of shift, so not all shift times perfectly aligned with 4-hour interval start and stop times. Thus, some intervals had overlap with more than one nurse shift. In this case, the nurse whose shift included the majority of the preceding interval was assigned.
3. In some cases, there were overlapping nurse shifts – that is, the end of an earlier shift was later than the start of a later shift. To select the correct nurse assignment, we followed the following steps:
   1. For all shifts <4 hours with any overlap with another nurse, the shorter (<4h) nurse shift was excluded, under an assumption that either this was a sign-in error, or alternatively, represented a very brief and likely inconsequential exposure time.
   2. For shifts >4 hours:
      - If the shift completely overlapped with one other nurse and both shifts are less than 14 hours, the shorter nurse shift (or a randomly selected shift if shift durations were identical) was excluded.
      - If the shift completely overlapped with one other nurse and one of the shifts starts at least 8 hours before the next shift, we changed the end time of the preceding nurse shift to the start time of the subsequent nurse shift, under an assumption that the first nurse did not sign out correctly.
      - If the shift overlapped with two other nurses (for example, the end of one other nurse’s shift and start of a second other nurse’s shift):
        - this nurse shift was dropped, if dropping would not create a gap, or
        - if a gap would be created, then this nurse’s shift was kept, and the start and end times of each overlapping shift was changed so that each shift end time was changed to equal the start time of the subsequent shift.

Attending physician assignments

Attending physician assignments relied on clinical notes as opposed to sign in data, which were known by the research team to be inaccurate. Attending physicians were routinely assigned by the day or week, with days starting at 7:00am. Therefore, we assigned physicians for 24-hour periods, or 6 four-hour intervals based on meta-data for History and Physical Exam and Progress Notes, including the author type (e.g., physician, anesthesiologist), service type (e.g., critical care, anesthesia), and the time stamp that the note was initiated. We found that each ICU had separate values for these variables that were relevant to identify attending physicians, based on staffing models. For example, some medical ICUs were staffed by Critical Care and Pulmonary physicians whereas some surgical ICUs were staffed by Critical Care, Trauma Surgery, and Anesthesiology physicians. These service line designations were among the meta data fields incorporated for determining assignment rules for each study ICU. These rules were compiled in a table of values and a prioritization scheme was determined for when multiple notes by different physicians were present. Notes written by residents or fellows were only used if co-signed by an identifiable attending physician. If a physician could not be identified, they were initially listed as “unassigned” for an entire 24-hour period (that is, 6 consecutive four-hour intervals). However, if the assignment on the day before and on the day after an unassigned period was the same physician, then the missing day was imputed to the flanking physician, based on clinical experience that physicians in most study ICUs worked at least a few consecutive days for continuity.

Value-add random assignment analysis

For random assignment analyses, clinician value-add was determined using combined models and were performed in the development dataset. First, we visually assessed distributional scatter plots of clinician value-add by patient LAPS2 at the time of first clinician assignment. We qualitatively assessed plots to determine discernible trends and quantitatively assessed linear trends using the Pearson correlation coefficient. Second, and consistent with previous clinical applications of VAM,(13) we assessed the statistical significance of clinician value-add using a linear model that regressed the initial LAPS2 score at the beginning of MV initiation onto NVA and PVA, using age, admission source, sex, admission Elixhauser comorbidity index score, admission LAPS2, medical vs surgical admission, hospital duration prior to ICU admission, and study ICU as covariates; in this model, variance was clustered at the encounter level.

| Clinician group | Partition | Mean | Standard deviation | Median | Interquartile range | Minimum | Maximum |
| --- | --- | --- | --- | --- | --- | --- | --- |
| Nurse | Development | -39 | 14 | -39 | [-47, -31] | -119 | 17 |
| Physician | Development | -38 | 16 | -39 | [-45, -31] | -131 | 14 |
| Nurse | Testing | -39 | 12 | -39 | [-46, -32] | -123 | 21 |
| Physician | Testing | -38 | 14 | -36 | [-46, -29] | -75 | 19 |

**Table E1** Summary statistics for marginal change in Laboratory Acute Physiology Score 2 based on combined models.

| **Covariate** | **Beta coefficient** | **95% CI** | **p-value** |
| --- | --- | --- | --- |
| NVA | 0.059 | (-0.010, 0.128) | 0.091 |
| PVA | 0.040 | (-0.082, 0.161) | 0.523 |
| Age | 0.143 | (0.069, 0.217) | <0.001 |
| Female sex | -1.907 | (-4.409, 0.595) | 0.135 |
| Elixhauser score | 0.201 | (0.136, 0.266) | <0.001 |
| Admission source (ref. ED admission) |  | | |
| Direct admit | -1.766 | (-5.568, 2.036) | 0.363 |
| Outside hospital transfer | -2.245 | (-5.320, 0.829) | 0.152 |
| Surgical admission (ref. medical admission) | -2.235 | (-5.174, 0.703) | 0.136 |
| Hospital duration prior to MV initiation | 0.027 | (0.018, 0.035) | <0.001 |
| Day of week (ref. Sunday) |  | | |
| Monday | -0.085 | \| (-0.509, 0.339) \| \| --- \| | 0.694 |
| Tuesday | 0.057 | \| (-0.501, 0.616) \| \| --- \| | 0.840 |
| Wednesday | 0.013 | \| (-0.582, 0.609) \| \| --- \| | 0.965 |
| Thursday | -0.205 | \| (-0.785, 0.376) \| \| --- \| | 0.489 |
| Friday | -0.179 | \| (-0.694, 0.336) \| \| --- \| | 0.495 |
| Saturday | 0.107 | \| (-0.304, 0.518) \| \| --- \| | 0.609 |
| Daytime shift (ref. night shift) | -0.100 | \| (-0.210, 0.009) \| \| --- \| | 0.073 |
| Admission LAPS2 | 0.374 | \| (0.343, 0.404) \| \| --- \| | <0.001 |
| Study ICU (ref. ICU 1) |  | | |
| 2 | 22.184 | \| (15.892, 28.475) \| \| --- \| | <0.001 |
| 3 | 1.914 | \| (-6.810, 10.637) \| \| --- \| | 0.667 |
| 4 | -8.875 | \| (-14.736, -3.014) \| \| --- \| | 0.003 |
| 5 | -13.367 | \| (-21.306, -5.428) \| \| --- \| | 0.001 |
| 6 | 8.805 | \| (2.338, 15.272) \| \| --- \| | 0.008 |
| 7 | -2.266 | \| (-11.258, 6.726) \| \| --- \| | 0.621 |
| 8 | -11.781 | \| (-20.703, -2.858) \| \| --- \| | 0.010 |
| 9 | 18.618 | \| (12.173, 25.063) \| \| --- \| | <0.001 |
| 10 | -12.123 | \| (-18.569, -5.676) \| \| --- \| | <0.001 |
| 11 | -13.147 | \| (-19.314, -6.980) \| \| --- \| | <0.001 |
| 12 | 4.172 | \| (-2.785, 11.129) \| \| --- \| | 0.24 |
| Constant | 84.146 | (75.519, 92.774) | <0.001 |

**Table E2.** Clinician value-add relationships with disease severity at mechanical ventilation initiation. Presented are model coefficients from a linear regression testing independent relationships between clinician value-add and disease severity at mechanical ventilation initiation as measured by the Laboratory Acute Physiology Score 2. CI- confidence interval; ED- emergency department; ICU- intensive care unit; LAPS2- Laboratory Acute Physiology Score 2; MV- mechanical ventilation; NVA- nurse value-add; PVA- physician value-add; Ref- reference variable.

| **Characteristic** | **All**^1^ | **Development**^1^ | **Test**^1^ |
| --- | --- | --- | --- |
| Change in LAPS2 during MV duration, median (IQR) | -37.8 (-36.0; 63.0) | -37.8 (-36.0; 62.0) | -37.8 (-37.0; 63.0) |
| Elixhauser score, median (IQR) | 22.7 (22.0; 27.0) | 22.5 (21.0; 27.0) | 22.8 (22.0; 26.0) |
| LAPS2 at admission, median (IQR) | 146.6 (145.0; 73.0) | 146.8 (145.0; 73.0) | 146.4 (145.0; 72.0) |
| Age, median (IQR) | 59.3 (61.0; 21.0) | 59.3 (61.0; 20.0) | 59.3 (61.0; 21.0) |
| Hospital duration prior to MV initiation, median hours (IQR) | 102.3 (11.5; 88.6) | 100.0 (11.4; 87.6) | 104.5 (11.6; 90.2) |
| Hospital length of stay, median hours (IQR) | 853.8 (622.8; 705.7) | 839.6 (619.3; 704.1) | 868.1 (626.9; 707.0) |
| ICU length of stay, median hours (IQR) | 486.6 (358.3; 465.4) | 478.4 (349.5; 456.1) | 494.8 (367.8; 471.6) |
| Mechanical ventilation duration, median hours (IQR) | 303.7 (156.2; 336.5) | 309.4 (156.1; 341.1) | 298.0 (156.4; 331.4) |
| Duration of time eligible for study inclusion, median hours (IQR) | 468.6 (341.6; 456.2) | 460.8 (333.8; 450.4) | 476.3 (351.9; 462.9) |
| Number of intervals, median (IQR) | 94.3 (85.0; 113.0) | 93.4 (83.0; 112.0) | 95.1 (87.0; 113.0) |
| Proportion of shifts that occurred during daytime, median (IQR) | 0.5 (0.5; 0.0) | 0.5 (0.5; 0.0) | 0.5 (0.5; 0.0) |
| Number of nurses per encounter, median (IQR) | 18.2 (17.0; 16.0) | 18.1 (17.0; 16.0) | 18.4 (18.0; 17.0) |
| Number of shifts per nurse, median (IQR) | 3.0 (3.0; 0.0) | 3.0 (3.0; 0.0) | 3.0 (3.0; 0.0) |
| Number of physicians per encounter, median (IQR) | 3.9 (4.0; 2.0) | 3.9 (4.0; 2.0) | 3.9 (4.0; 2.0) |
| Number of intervals per physician, median (IQR) | 5.8 (6.0; 0.0) | 5.8 (6.0; 0.0) | 5.8 (6.0; 0.0) |
| Proportion medical admissions, n (%) | 328,362.0 (37.9%) | 165,102.0 (38.2%) | 163,260.0 (37.5%) |
| Proportion surgical admissions, n (%) | 538,862.0 (62.1%) | 267,268.0 (61.8%) | 271,594.0 (62.5%) |
| Female sex | 352,778.0 (40.7%) | 176,983.0 (40.9%) | 175,795.0 (40.4%) |
| Race |  |  |  |
| American Indian or Alaskan Native, n (%) | 1,554.0 (0.2%) | 908.0 (0.2%) | 646.0 (0.1%) |
| Asian, n (%) | 28,943.0 (3.3%) | 14,513.0 (3.4%) | 14,430.0 (3.3%) |
| Black or African American, n (%) | 294,653.0 (34.0%) | 147,004.0 (34.0%) | 147,649.0 (34.0%) |
| More Than One Race, n (%) | 11,479.0 (1.3%) | 5,754.0 (1.3%) | 5,725.0 (1.3%) |
| Native Hawaiian or Other Pacific Islander, n (%) | 849.0 (0.1%) | 598.0 (0.1%) | 251.0 (0.1%) |
| Unknown or Not Reported, n (%) | 94,631.0 (10.9%) | 46,705.0 (10.8%) | 47,926.0 (11.0%) |
| White, n (%) | 435,115.0 (50.2%) | 216,888.0 (50.2%) | 218,227.0 (50.2%) |
| Admission source |  |  |  |
| ED admissions, n (%) | 346,975.0 (40.0%) | 175,612.0 (40.6%) | 171,363.0 (39.4%) |
| Direct admissions, n (%) | 161,434.0 (18.6%) | 79,118.0 (18.3%) | 82,316.0 (18.9%) |
| Outside facility transfers, n (%) | 358,815.0 (41.4%) | 177,640.0 (41.1%) | 181,175.0 (41.7%) |
| Ethnicity |  |  |  |
| Hispanic, n (%) | 31,335.0 (3.6%) | 15,480.0 (3.6%) | 15,855.0 (3.6%) |
| Not Hispanic, n (%) | 796,157.0 (91.8%) | 396,821.0 (91.8%) | 399,336.0 (91.8%) |
| Unknown, n (%) | 39,732.0 (4.6%) | 20,069.0 (4.6%) | 19,663.0 (4.5%) |

**Table E3. Baseline characteristics of imputation dataset.** ^1^Mean (Median; IQR); n (%).

|  | Base model | Nurse model | Physician model | Combined model |
| --- | --- | --- | --- | --- |
| R^2^ | 0.12 | 0.17 | 0.16 | 0.19 |
| Adjusted R^2^ | 0.12 | 0.17 | 0.16 | 0.18 |
| AIC | 4,516,957 | 4,494,706 | 4,498,779 | 4,488,203 |

**Table E4.** **Imputed model performance.** All models were built using the imputation approach described in the text. Base models were adjusted for patient age, gender, admission Elixhauser score, admission source, medical vs surgical admission, hospital duration prior to ICU admission, day of week, LAPS2 at ICU admission, and LAPS2 on day of service. Physician model contained the same variables as the base model and was additionally adjusted for physician assignments. Nurse model contained the same variables as the base model and was additionally adjusted for physician assignments. The combined model contained the same variables as the base model and was additionally adjusted for both physician and nurse assignments. AIC- Akaike information criterion, R^2^- coefficient of determination.

**Figure legends**

**Figure E1.** Correlation of nurse-associated *ΔLAPS* between development and test partitions. These graphs plot the predicted clinician value using the combined model across and paired between the development and test partitions. **E1.** Paired predicted nurse value. **E2.** Paired predicted physician value. For both correlation coefficients, p<0.001.

**Figure E2.** Correlation of clinician value between development and test sets. These graphs plot the predicted clinician value using the combined model across and paired between the development and test sets. **E1.** Paired predicted nurse value. **E2.** Paired predicted physician value. For both correlation coefficients, p<0.001.

**Figure E3A.** Distribution of nurse-associated LAPS2 score at the time of first nurse assignment. LAPS2- Laboratory acute physiology score 2; *ΔLAPS*-change in Laboratory Acute Physiology Score 2 from the beginning to end of intensive care unit admission.

**Figure E3B.** Distribution of physician-associated LAPS2 score at the time of first physician assignment. LAPS2- Laboratory acute physiology score 2; *ΔLAPS*-change in Laboratory Acute Physiology Score 2 from the beginning to end of intensive care unit admission.

**Figure E4A.** Frequency distribution of nurse associated change in disease severity during intensive care unit admission in the missingness sensitivity analysis. Top panel: frequency distribution in development partition. Bottom panel: frequency distribution in testing partition. *ΔLAPS*-change in Laboratory Acute Physiology Score 2 from the beginning to end of intensive care unit admission.

**Figure E4B.** Frequency distribution of physician associated change in disease severity during intensive care unit admission in the missingness sensitivity analysis. Top panel: frequency distribution in development partition. Bottom panel: frequency distribution in testing partition. *ΔLAPS*-change in Laboratory Acute Physiology Score 2 from the beginning to end of intensive care unit admission.

**Figure E5A.** Correlation of nurse-associated *ΔLAPS* between development and test partitions in the missingness sensitivity analysis. These graphs plot the predicted clinician value using the combined model across and paired between the development and test partitions. **2A.** Paired predicted nurse value. **2B.** Paired predicted physician value. For both correlation coefficients, p<0.001.

**Figure E5B.** Correlation of clinician value between development and test sets in the missingness sensitivity analysis. These graphs plot the predicted clinician value using the combined model across and paired between the development and test sets. **2A.** Paired predicted nurse value. **2B.** Paired predicted physician value. For both correlation coefficients, p<0.001.

**Figure E6A.** Distribution of nurse-associated LAPS2 score at the time of first nurse assignment in the missingness sensitivity analysis. LAPS2- Laboratory acute physiology score 2; *ΔLAPS*-change in Laboratory Acute Physiology Score 2 from the beginning to end of intensive care unit admission.

**Figure E6B.** Distribution of physician-associated LAPS2 score at the time of first physician assignment in the missingness sensitivity analysis. LAPS2- Laboratory acute physiology score 2; *ΔLAPS*-change in Laboratory Acute Physiology Score 2 from the beginning to end of intensive care unit admission.

**Figure E7A.** Frequency distribution of nurse associated change in disease severity during intensive care unit admission in the first 7 days of mechanical ventilation. Top panel: frequency distribution in development partition. Bottom panel: frequency distribution in testing partition. *ΔLAPS*-change in Laboratory Acute Physiology Score 2 from the beginning to end of intensive care unit admission.

**Figure E7B.** Frequency distribution of physician associated change in disease severity during intensive care unit admission in the first 7 days of mechanical ventilation. Top panel: frequency distribution in development partition. Bottom panel: frequency distribution in testing partition. *ΔLAPS*-change in Laboratory Acute Physiology Score 2 from the beginning to end of intensive care unit admission.

**Figure E8A.** Correlation of nurse-associated *ΔLAPS* between development and test partitions in the first 7 days of mechanical ventilation. These graphs plot the predicted clinician value using the combined model across and paired between the development and test partitions. **8A.** Paired predicted nurse value. **8B.** Paired predicted physician value. For both correlation coefficients, p<0.001.

**Figure E8B.** Correlation of nurse-associated *ΔLAPS* between development and test partitions in the first 7 days of mechanical ventilation. These graphs plot the predicted clinician value using the combined model across and paired between the development and test partitions. **8A.** Paired predicted nurse value. **8B.** Paired predicted physician value. For both correlation coefficients, p<0.001.
